# Supplementary figures and images for: Statistical correction of the Winner’s Curse explains replication variability in quantitative trait genome-wide association studies
Source: PLoS Genet. 2017 Jul 17;13(7):e1006916. doi: 10.1371/journal.pgen.1006916 (PMC5536394; doi:10.1371/journal.pgen.1006916)

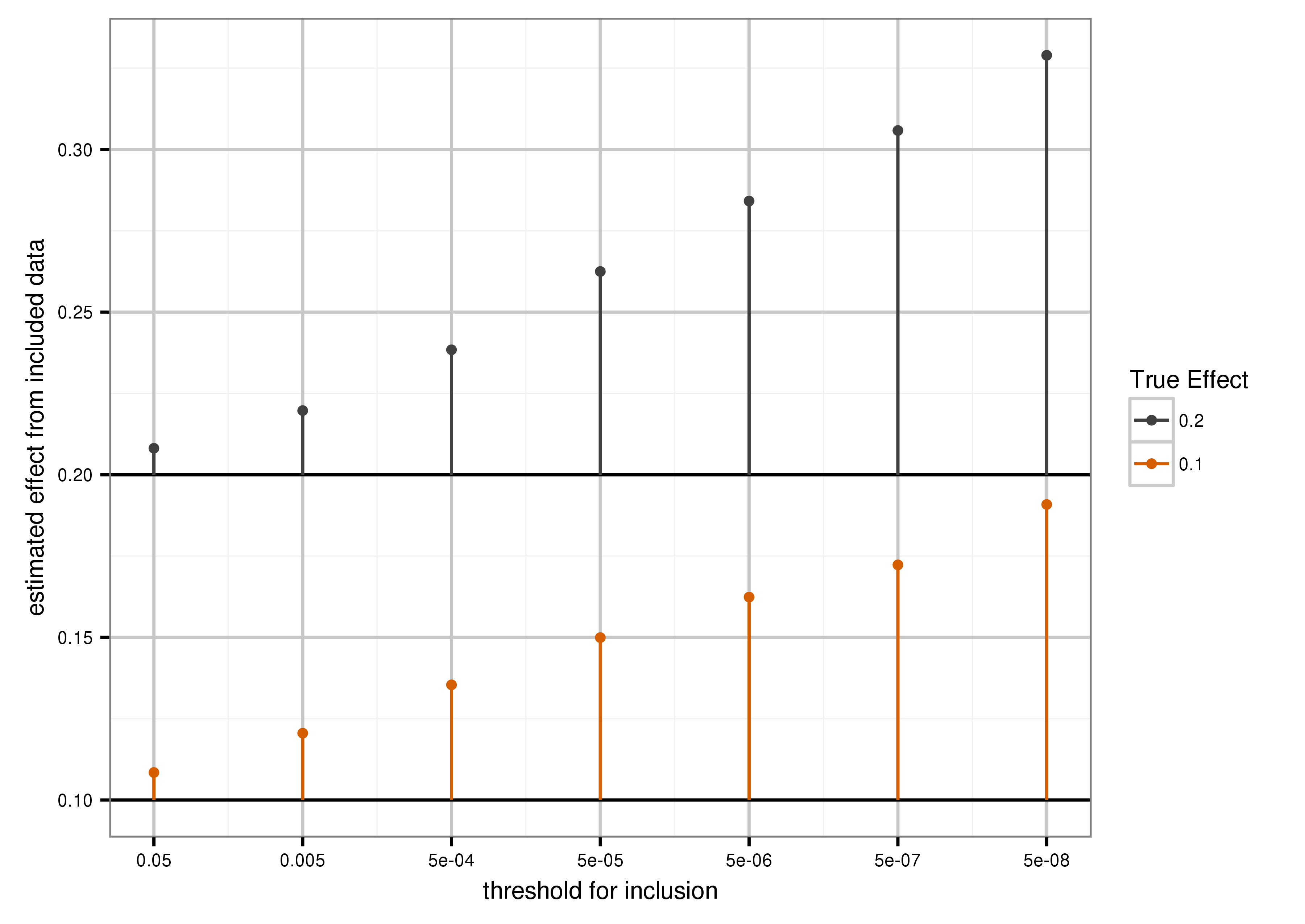

Supplement: S1 Fig — A SNP with fixed frequency, sample size, and phenotypic variance explained was repeatedly drawn at random, and the accompanying standard normal trait was simulated. The apparent effect of SNP on trait was estimated exclusively from simulations exceeding progressively more stringent replication α. X-axis: replication α; Y-axis: estimated SNP effect from linear regression. Two simulations with different effect estimates (horizontal lines) are shown. The height of the vertical lines corresponds to the average bias (curse) introduced by discovery prioritization. (TIFF) [file pgen.1006916.s001.tiff]

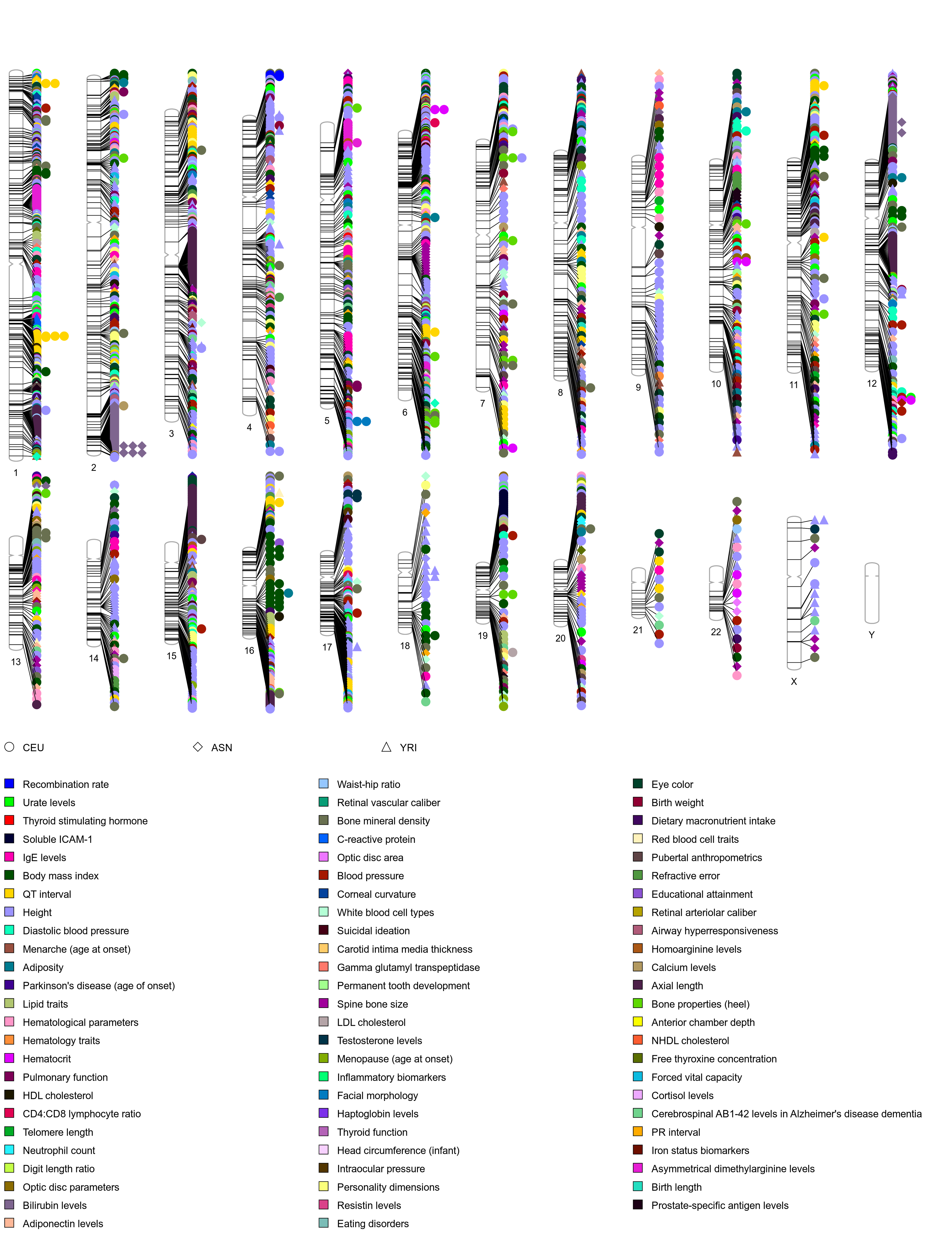

Supplement: S2 Fig — Includes all loci brought forward from discovery, not filtered for independence. Datapoint shapes correspond to approximate continental ancestry: CEU = European, YRI = (sub-Saharan West African, ASN = East Asian. Individual sites are stacked horizontally when the same variant is tested in multiple contexts. Distribution of variants across chromosomes is nonrandom relative to distribution of SNPs in reference datasets (p < 0.00001). Generated using the software at [26]. (TIFF) [file pgen.1006916.s002.tiff]

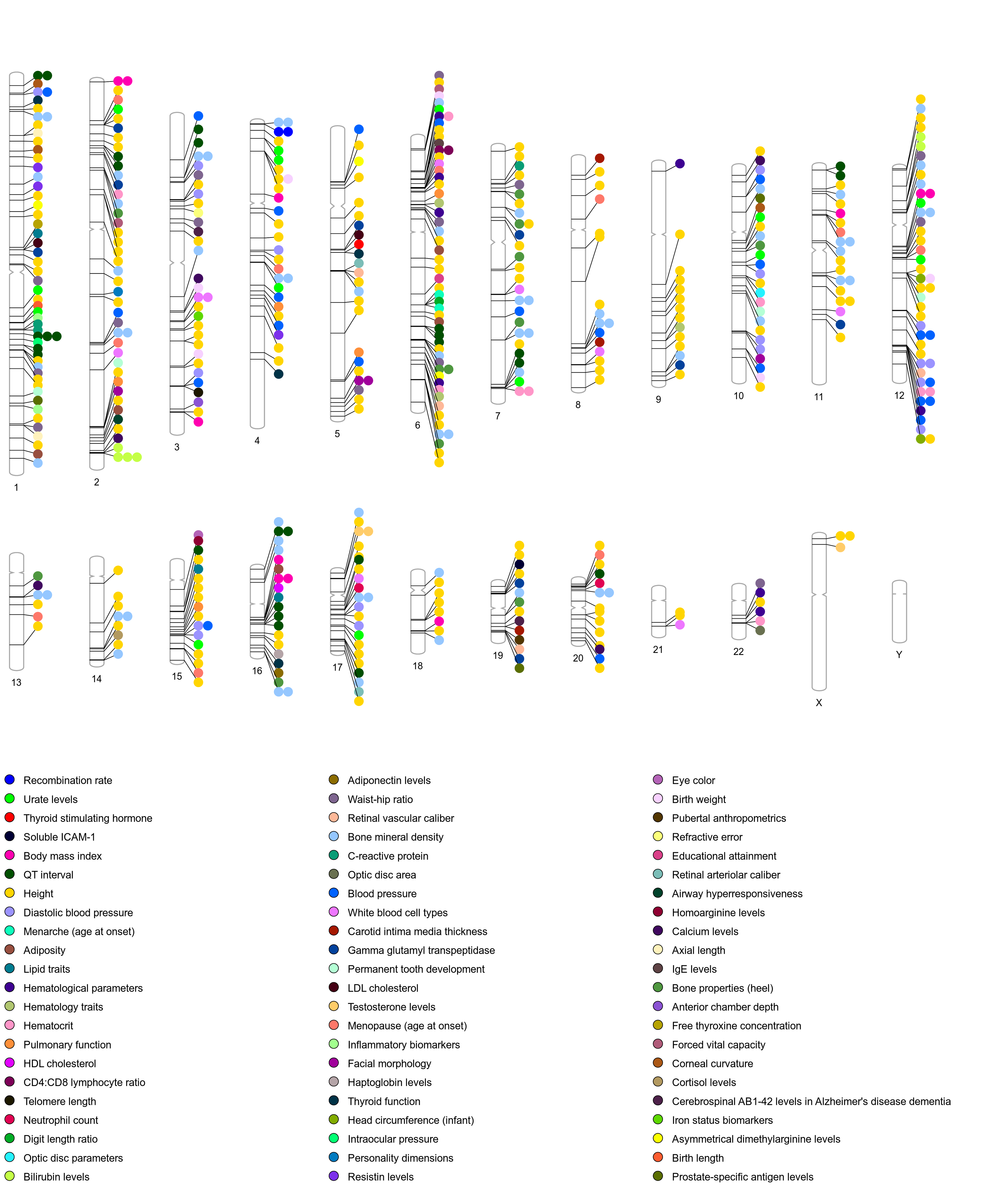

Supplement: S3 Fig — Subset of data from Supp. Fig; includes only loci passing study-specific Bonferroni-corrected replication threshold. Generated using the software at [26]. (TIFF) [file pgen.1006916.s003.tiff]

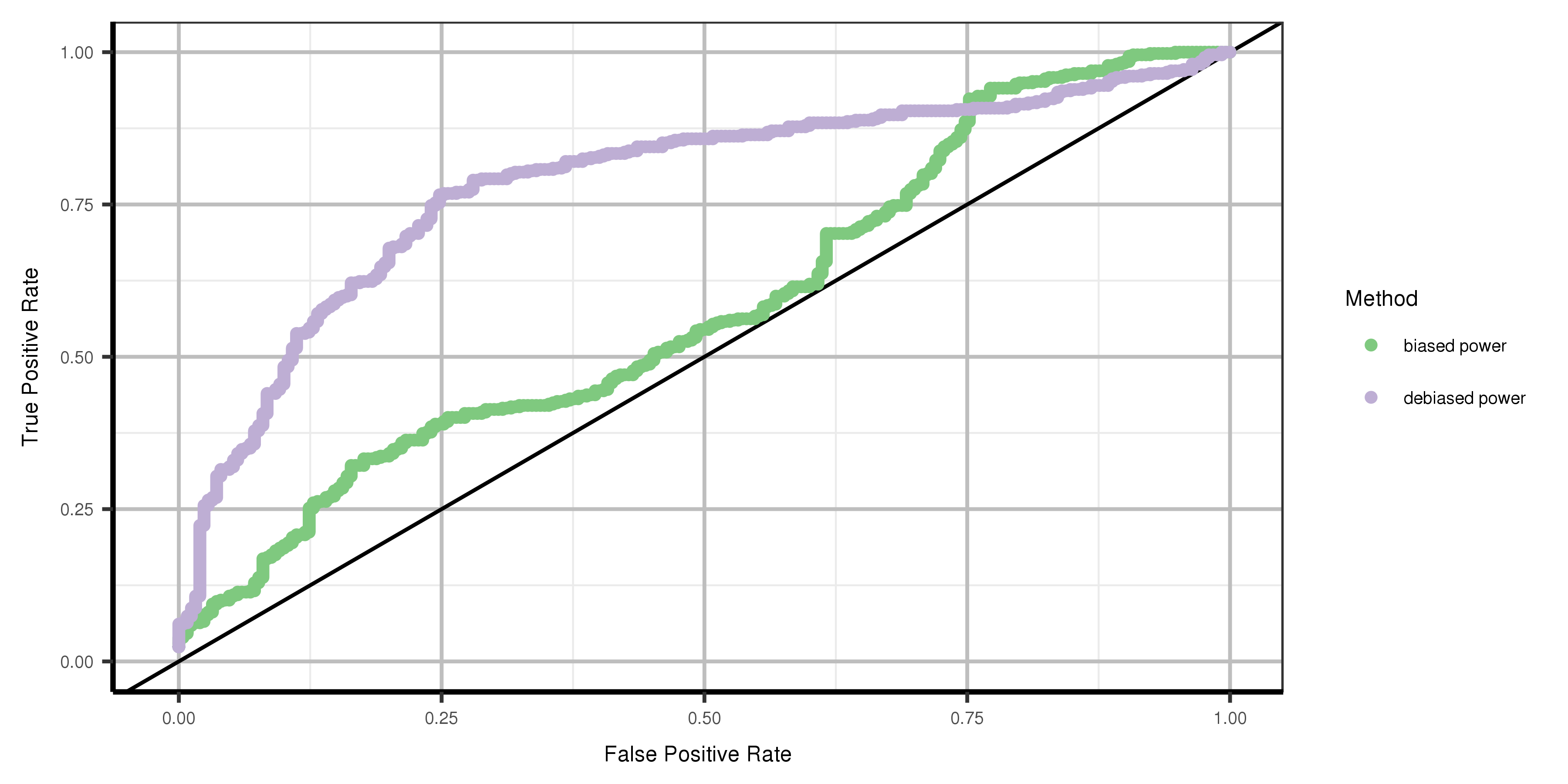

Supplement: S4 Fig — Plotted are receiver operating characteristics (ROC) for raw discovery and WC-corrected estimates of power to nominally replicate variants in individual studies. X-axis: false positive rate, computed as number of non-replicating variants having greater than a given power to replicate; Y-axis: true positive rate, computed as number of replicating variants having such power. AUC: 0.795 (WC-corrected) versus 0.579 (biased); DeLong’s two-tailed p < 2 ⋅ 10−16. Considers studies with same ancestry in both discovery and replication and correct per-locus sample sizes, as used in the main results. (TIFF) [file pgen.1006916.s004.tiff]

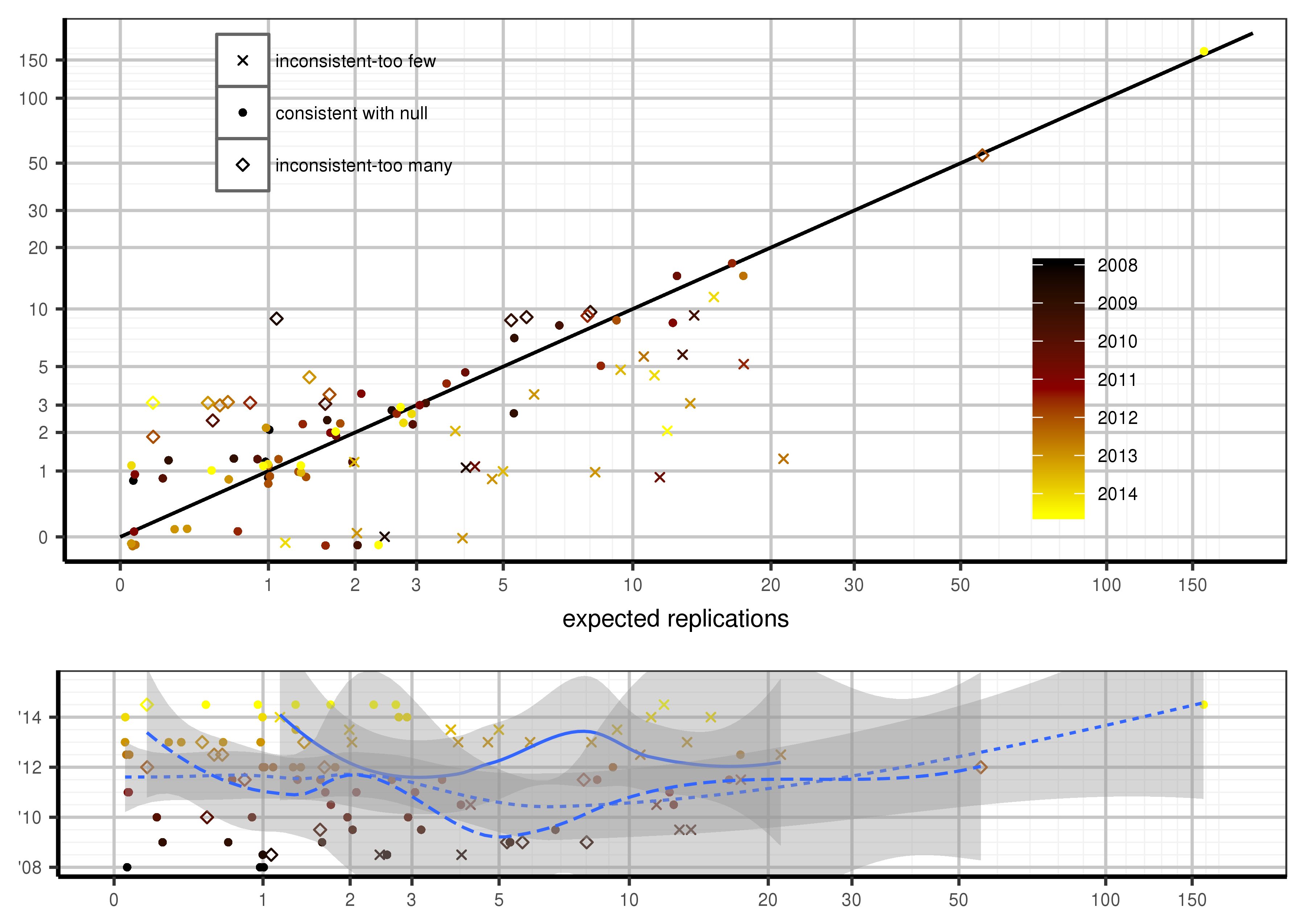

Supplement: S5 Fig — Top panel: predicted versus expected replication for each paper, with each paper flagged as being within 95% confidence of predicted replication rate under WC-corrected model, greater than or less than expectation. X-axis: predicted number of replications in a given paper, calculated as the sum across all loci of power to replicate based on WC-corrected discovery effect estimates. Y-axis: observed number of replications in the paper. Colors correspond to publication dates binned into six month intervals, from 2008 to 2014. Point shapes to correspondence between observed and expected rates. Replication is defined as a one-tailed replication p-value surpassing a per-paper Bonferroni threshold: 0.05#loci per paper. Confidence intervals defined as 95% confidence according to Poisson binomial draws from the WC-corrected power distribution. Bottom panel: papers underperforming the WC-corrected model tend to have been published later; papers overperforming the WC-corrected model tend to have been published earlier. While these trends are significant, they are dominated by the large number of QC-passing papers from Nature Genetics, which possess an earlier average publication date as well as a replication rate significantly higher than expected based on reported power. (TIFF) [file pgen.1006916.s005.tiff]

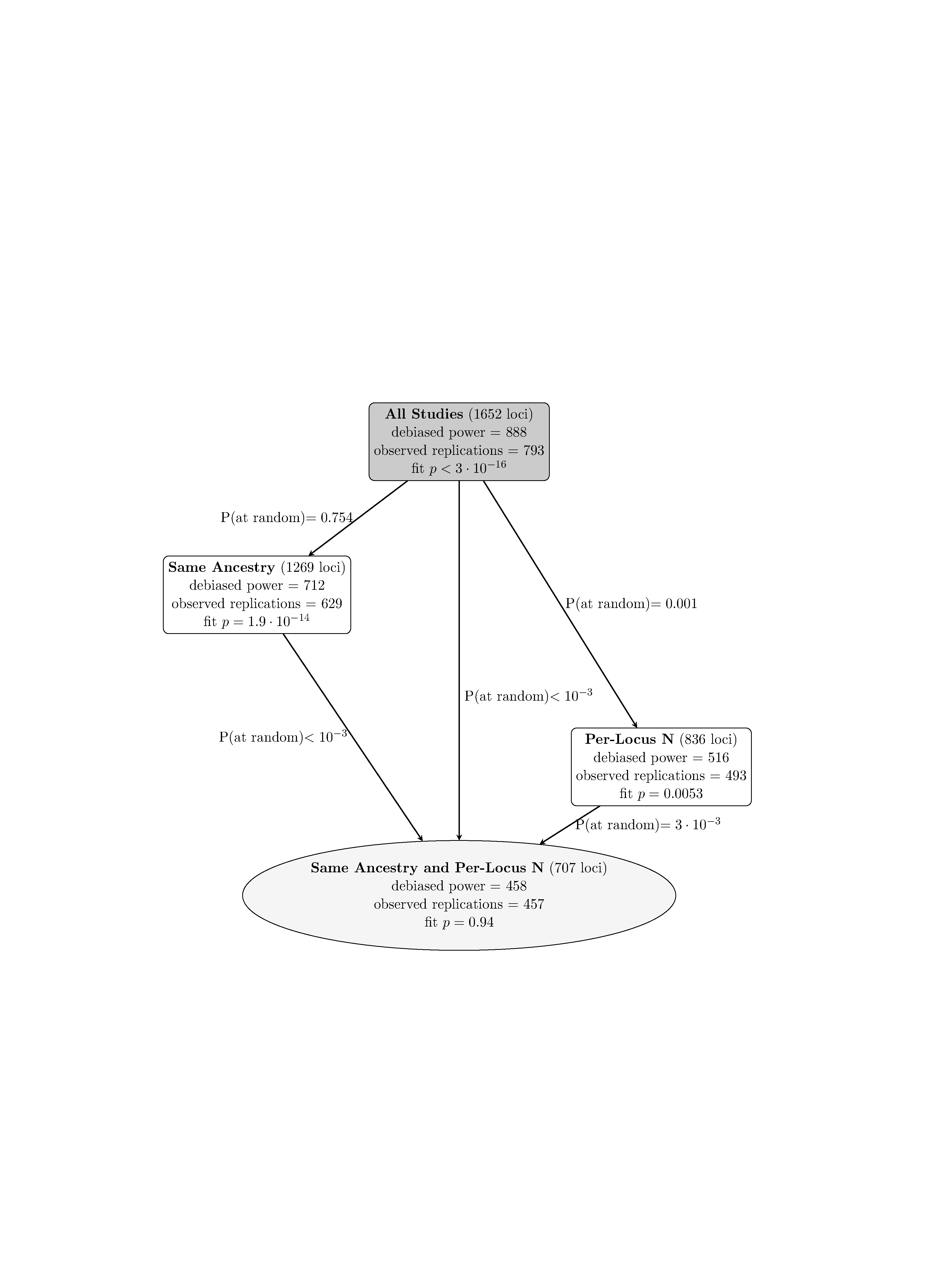

Supplement: S6 Fig — Each node corresponds to one method of subsetting the data: all papers; papers conducting discovery and replication in the same continental ancestry; papers reporting correct per-locus sample sizes; or papers doing both. Probabilities in nodes correspond to two-tailed Poisson binomial fit test for prediction of nominal replication rates after Winner’s Curse correction. Probabilities along edges correspond to the chance of randomly seeing improvement of fit between connected nodes at least as large as observed due to loss of power exclusively, based on 10000 simulated subsamplings from the source node matched on total predicted power to replicate. Relative position of nodes along vertical axis corresponds to number of loci removed in the subset. (TIFF) [file pgen.1006916.s006.tiff]

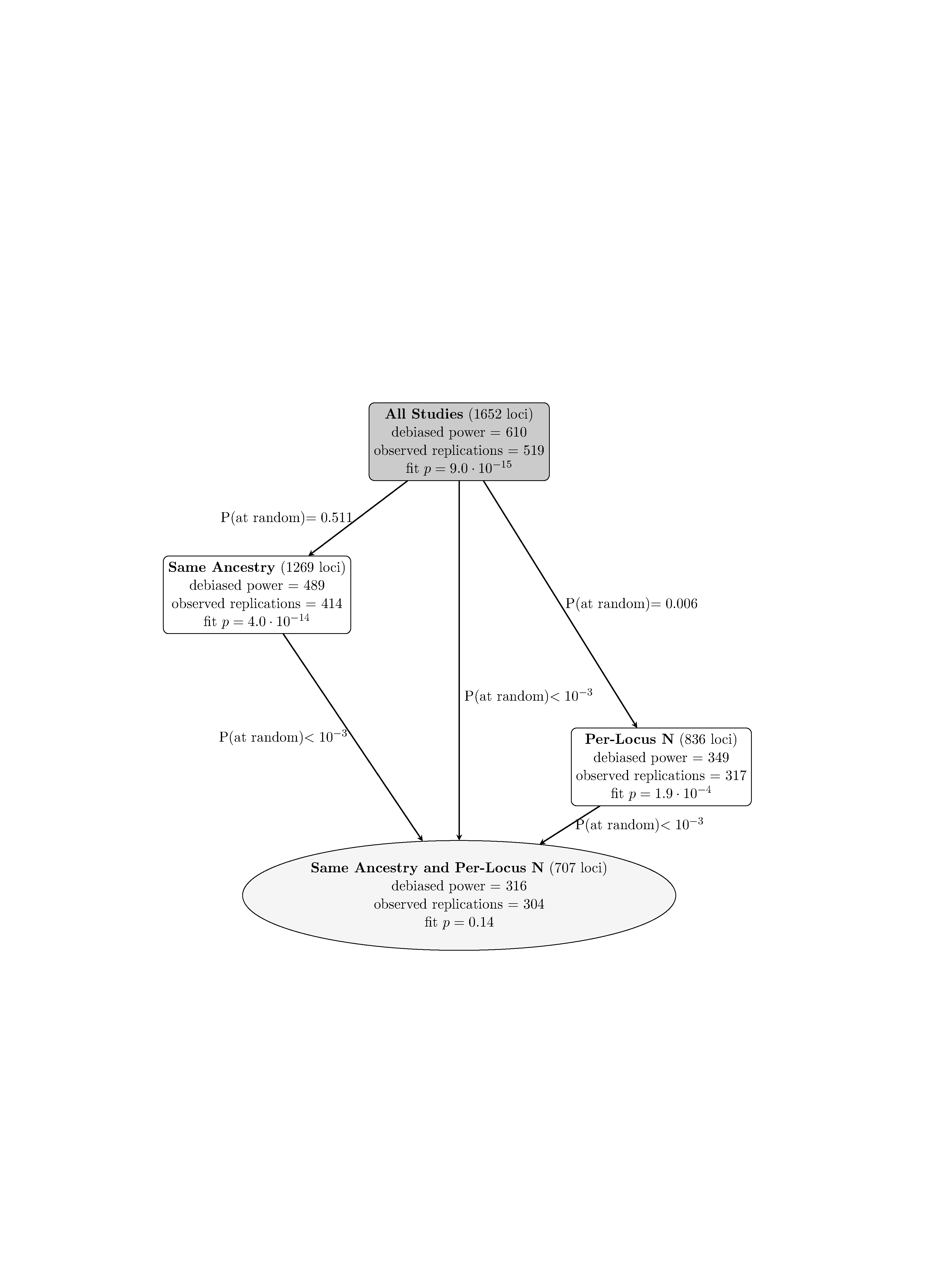

Supplement: S7 Fig — Each node corresponds to one method of subsetting the data: all papers; papers conducting discovery and replication in the same continental ancestry; papers reporting correct per-locus sample sizes; or papers doing both. Probabilities in nodes correspond to two-tailed Poisson binomial fit test for prediction of Bonferroni replication rates after Winner’s Curse correction. Probabilities along edges correspond to the chance of randomly seeing improvement of fit between connected nodes at least as large as observed due to loss of power exclusively, based on 10000 simulated subsamplings from the source node matched on total predicted power to replicate. Relative position of nodes along vertical axis corresponds to number of loci removed in the subset. (TIFF) [file pgen.1006916.s007.tiff]

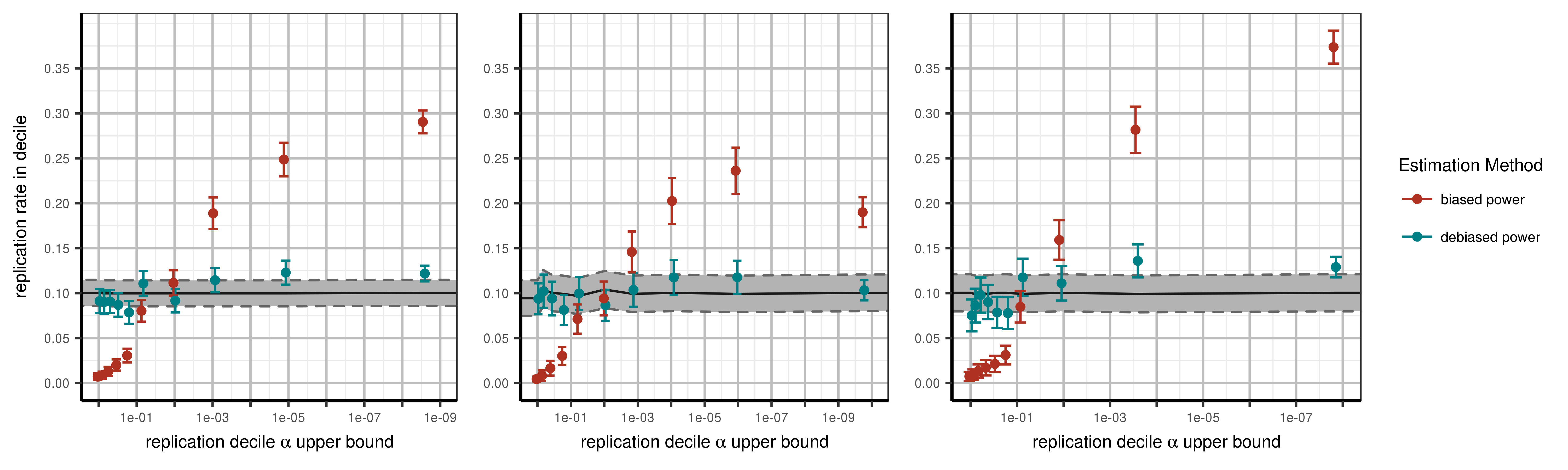

Supplement: S8 Fig — X-axis: left boundary of bin on replication threshold; Y-axis: percentage of replication within corresponding bin. Tracks correspond to actual data in the replication study, predicted power to replicate using discovery effect estimates, and predicted power to replicate using WC-corrected discovery effect estimates. Loci brought forward to replication in papers were pruned to contain independent signals within (but not necessarily between) individual papers. Error bars correspond to 95% confidence intervals around mean replication rates as estimated across multiple loci. Left panel: including all papers; middle panel: including only papers with accurate per-locus sample sizes; right panel: including only papers with maximal sample sizes per variant. Including only maximum sample size regardless of per-variant missingness will uniformly inflate replication rate estimates. (TIFF) [file pgen.1006916.s008.tiff]

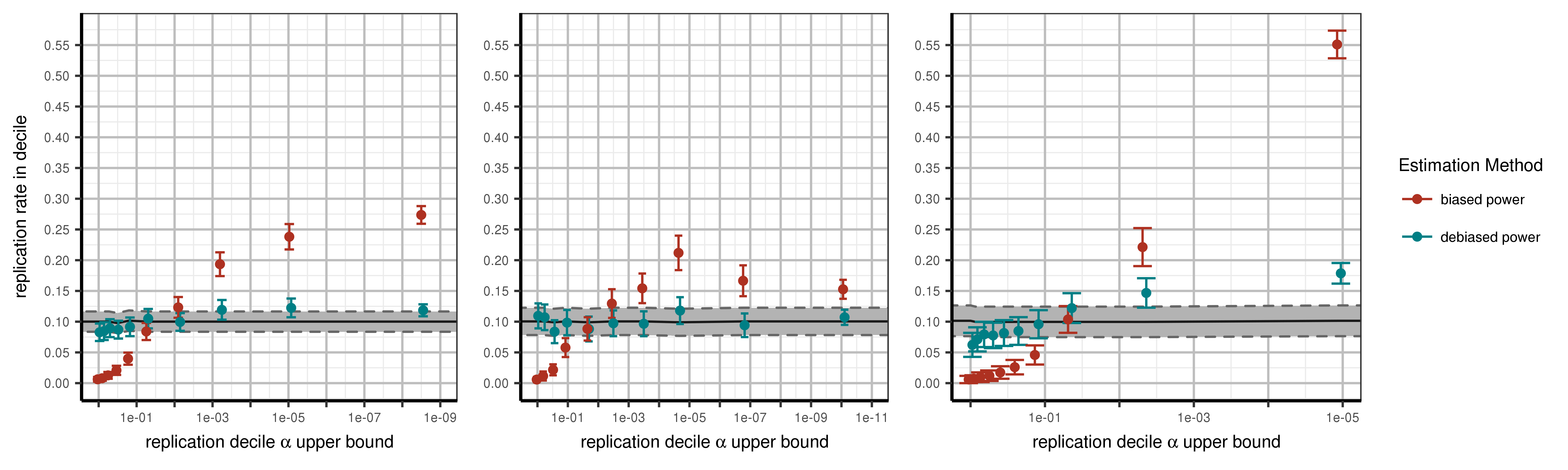

Supplement: S9 Fig — X-axis: left boundary of bin on replication threshold; Y-axis: percentage of replication within corresponding bin. Tracks correspond to actual data in the replication study, predicted power to replicate using discovery effect estimates, and predicted power to replicate using WC-corrected discovery effect estimates. Loci brought forward to replication in papers were pruned to contain independent signals within (but not necessarily between) individual papers. Error bars correspond to 95% confidence intervals around mean replication rates as estimated across multiple loci. Left panel: including all papers; middle panel: including only papers with accurate per-locus sample sizes; right panel: including only papers with maximal sample sizes per variant. Including only maximum sample size regardless of per-variant missingness will uniformly inflate replication rate estimates. (TIFF) [file pgen.1006916.s009.tiff]

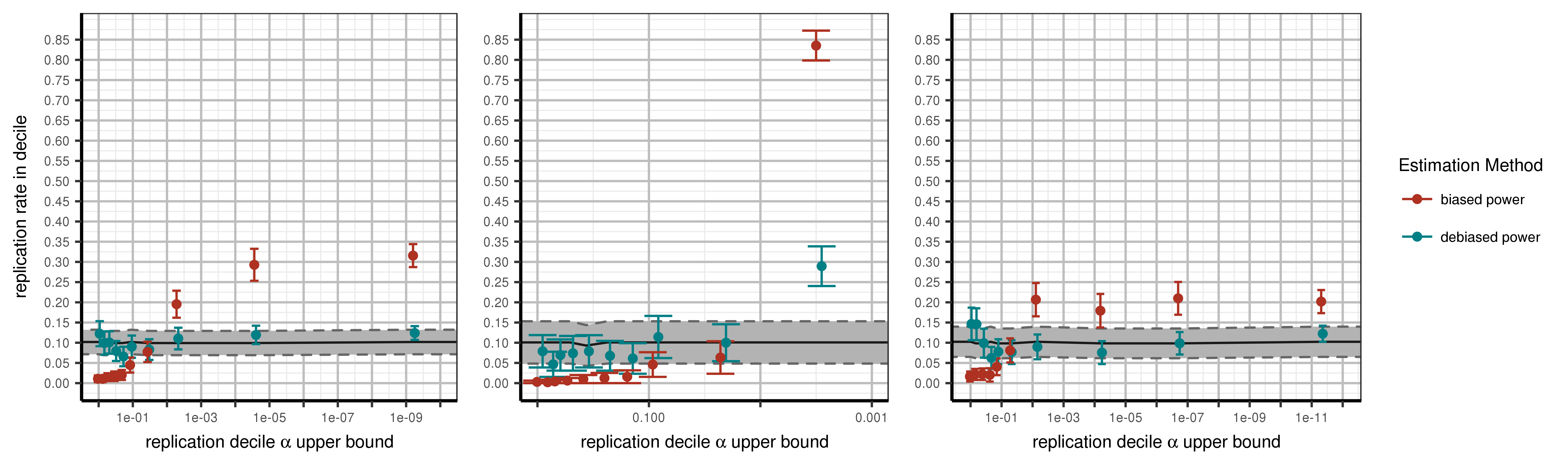

Supplement: S10 Fig — X-axis: left boundary of bin on replication threshold; Y-axis: percentage of replication within corresponding bin. Tracks correspond to actual data in the replication study, predicted power to replicate using discovery effect estimates, and predicted power to replicate using WC-corrected discovery effect estimates. Loci brought forward to replication in papers were pruned to contain independent signals within (but not necessarily between) individual papers. Error bars correspond to 95% confidence intervals around mean replication rates as estimated across multiple loci. Left panel: including all papers; middle panel: including only papers with accurate per-locus sample sizes; right panel: including only papers with maximal sample sizes per variant. Including only maximum sample size regardless of per-variant missingness will uniformly inflate replication rate estimates. (TIFF) [file pgen.1006916.s010.tiff]

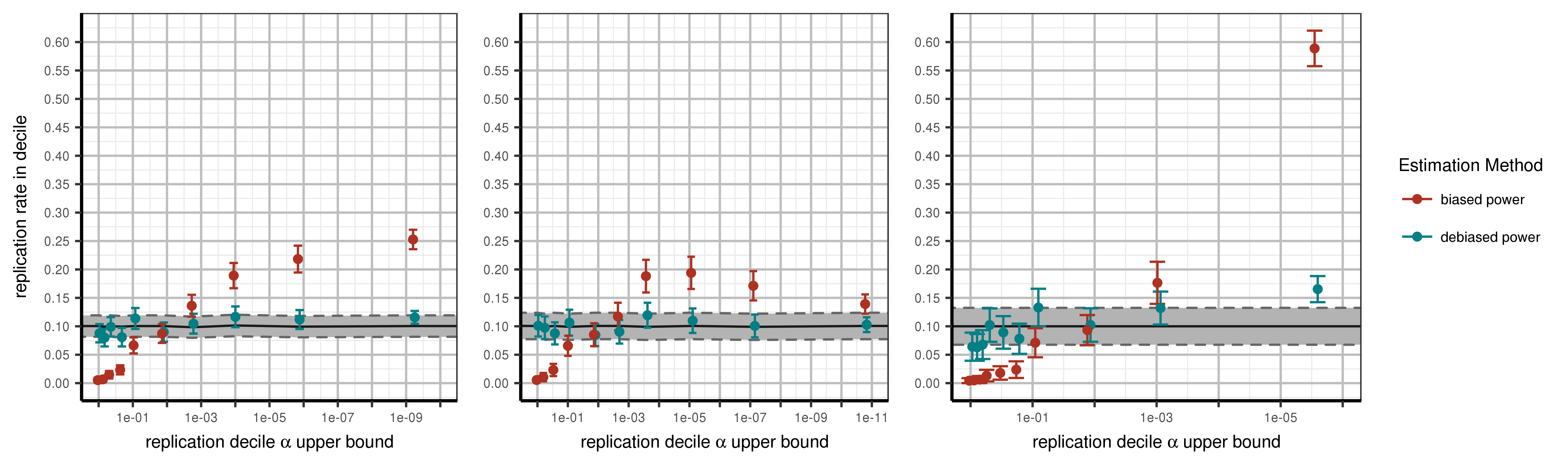

Supplement: S11 Fig — X-axis: left boundary of bin on replication threshold; Y-axis: percentage of replication within corresponding bin. Tracks correspond to actual data in the replication study, predicted power to replicate using discovery effect estimates, and predicted power to replicate using WC-corrected discovery effect estimates. Loci brought forward to replication in papers were pruned to contain independent signals within (but not necessarily between) individual papers. Error bars correspond to 95% confidence intervals around mean replication rates as estimated across multiple loci. Left panel: including all papers; middle panel: including only papers with accurate per-locus sample sizes; right panel: including only papers with maximal sample sizes per variant. Including only maximum sample size regardless of per-variant missingness will uniformly inflate replication rate estimates. (TIFF) [file pgen.1006916.s011.tiff]
