# Supplementary material for: Statistical correction of the Winner’s Curse explains replication variability in quantitative trait genome-wide association studies
Source: PLoS Genet. 2017 Jul 17;13(7):e1006916. doi: 10.1371/journal.pgen.1006916 (PMC5536394; doi:10.1371/journal.pgen.1006916)
Supplement: S1 File — Contains: Tables A-C, and bibliography for all papers considered from the NHGRI-EBI GWAS Catalog. (PDF) [file pgen.1006916.s012.pdf]

Supplement for: Statistical Correction of the Winner's Curse Explains Replication Variability in Human Quantitative Trait GWAS

Cameron Palmer[1,*], Itsik Pe'er[1,2]

1 Department of Systems Biology, Columbia University Medical Center, New York, New York, United States of America
2 Department of Computer Science, Columbia University, New York, New York, United States of America

* cdp2130@cumc.columbia.edu

Contents

Contents **1**

References **5**

	Analyzed	Filtered
Am J Hum Genet	8	23
Am J Med Genet B Neuropsychiatr Genet	2	2
Ann Hum Genet	0	1
BMC Genomics	0	1
BMC Med Genet	3	3
BMC Med Genomics	0	1
Circ Cardiovasc Genet	4	12
Eur J Hum Genet	0	3
Front Genet	1	0
G3 (Bethesda)	0	1
Gene	1	1
Genes Brain Behav	0	1
Genes Immun	0	1
Genet Epidemiol	1	1
Genomics	0	1
Genomics Inform	0	1
Hum Genet	3	7
Hum Mol Genet	24	48
Immunogenetics	0	2
J Hum Genet	0	2
J Med Genet	1	5
Nat Genet	26	48
Nature	0	7
PLoS Genet	18	31
PLoS One	6	21
Pharmacogenet Genomics	0	1
Pharmacogenomics	0	1
Pharmacogenomics J	0	1
Science	2	2
Twin Res Hum Genet	0	3
Total:	100	232

Table A. Distribution of quantitative trait GWAS papers across journals, for journals that had at least one article annotated as "attempting replication" in the NHGRI-EBI GWAS Database as of 04 Feb 2016.

Filter type	Filter subcategories	Papers removed	Papers remaining
Quantitative trait studies in GWAS Catalog		691	332
Complete data reporting for study	all attempted replications reported	121	211
Threshold replication model		31	180
Complete data reporting for variants	trait mean, variant frequency, sample size, effect size, standard error or p-value	70	110
Statistical test restrictions	frequentist, additive, same trait in both stages, etc.	10	100

Table B. Summary of quality control process applied to 332 candidate quantitative trait GWAS papers. Filters are hierarchical, in the sense that a paper failing a criterion at one stage of the process was not evaluated for other criteria of lower priority. The 100 papers passing these filters were further annotated for other discrepancies that would interfere with but not entirely prevent debiasing calculations: allele frequency from reference population instead of actual cohort; allele frequency from one round but not both; maximum sample size reported instead of per-variant sample size, reflecting missingness; extreme low precision errors; etc.

Ancestry	N Reported	Papers	Loci	PB p	1	2	3	4	5	6	7	8	9	10
All	all	100	1652	$<$ .0001	.17	.15	.18	.058	.0014	.15	.22	.038	5.6E-4	7.4E-07
	per-locus N	43	836	.0053	.95	.75	.49	.058	.86	.061	.71	.098	.057	.69
	max N	57	816	$<$ .0001	.0072	.17	.81	.35	.02	.017	.1	.32	6.5E-5	1.8E-06
Same	all	87	1269	$<$ .0001	.02	.042	.16	.16	.21	.6	.92	.018	.0037	2.8E-04
	per-locus N	39	707	.94	.41	.54	.13	.84	.3	.79	.74	.095	.51	.33
	max N	48	562	$<$ .0001	2.1e-4	.011	.075	.051	.11	.22	.77	.091	1.4E-4	2.0E-15
Different	all	13	383	.043	.12	1.0	1.0	.15	.012	.41	.24	.45	.12	.0089
	per-locus N	4	129	$<$ .0001	.88	.0082	.15	.25	.5	.15	.063	.78	1.0	3.0E-16
	max N	9	254	.019	.018	.022	1.0	.012	.23	.17	.45	.13	1.0	.057
European	all	60	976	$<$ .0001	.14	.019	.86	.033	.19	.22	.54	.11	.16	.0079
	per-locus N	29	646	.371	1.0	.88	.21	.59	.14	.35	.09	.45	.94	.82
	max N	31	330	$<$ .0001	.022	.0068	.023	1.0	.49	.14	.076	1.0	.045	7.3E-09

Table C. Probability of observing true replication counts within decile bins according to WC-corrected power estimates. Data are partitioned by ancestry matching between discovery and replication as well as reporting of per-locus or max sample size. First three data columns are: number of papers in category, number of (independent) loci in category, Poisson binomial fit two-sided p-value for nominal replication in subset of papers. For graphical example of decile bins, see Fig 2. Null hypothesis for decile p-values: power within decile according to WC-corrected replication effect estimates is true generative probability for each variant, according to Poisson binomial distribution (Methods). Bins for which null is likely false (Poisson binomial two-sided test $p < 0.01$) are shaded.

Citations for Supplement and All Analyzed Papers

References

1. Wolfe, D., Dudek, S., Ritchie, M. D. & Pendergrass, S. A. Visualizing genomic information across chromosomes with PhenoGram. *BioData Min* **6**, 18 (2013).

2. Kong, A. *et al.* Sequence variants in the RNF212 gene associate with genome-wide recombination rate. *Science* **319**, 1398–401 (2008).

3. Vitart, V. *et al.* SLC2A9 is a newly identified urate transporter influencing serum urate concentration, urate excretion and gout. *Nat. Genet.* **40**, 437–42 (2008).

4. Arnaud-Lopez, L. *et al.* Phosphodiesterase 8B gene variants are associated with serum TSH levels and thyroid function. *Am. J. Hum. Genet.* **82**, 1270–80 (2008).

5. Paré, G. *et al.* Novel association of ABO histo-blood group antigen with soluble ICAM-1: results of a genome-wide association study of 6,578 women. *PLoS Genet.* **4**, e1000118 (2008).

6. Weidinger, S. *et al.* Genome-wide scan on total serum IgE levels identifies FCER1A as novel susceptibility locus. *PLoS Genet.* **4**, e1000166 (2008).

7. Thorleifsson, G. *et al.* Genome-wide association yields new sequence variants at seven loci that associate with measures of obesity. *Nat. Genet.* **41**, 18–24 (2009).

8. Newton-Cheh, C. *et al.* Common variants at ten loci influence QT interval duration in the QTGEN Study. *Nat. Genet.* **41**, 399–406 (2009).

9. Pfeufer, A. *et al.* Common variants at ten loci modulate the QT interval duration in the QTSCD Study. *Nat. Genet.* **41**, 407–14 (2009).

10. Soranzo, N. *et al.* Meta-analysis of genome-wide scans for human adult stature identifies novel Loci and associations with measures of skeletal frame size. *PLoS Genet.* **5**, e1000445 (2009).

11. Levy, D. *et al.* Genome-wide association study of blood pressure and hypertension. *Nat. Genet.* **41**, 677–87 (2009).

12. Newton-Cheh, C. *et al.* Genome-wide association study identifies eight loci associated with blood pressure. *Nat. Genet.* **41**, 666–76 (2009).

13. Sulem, P. *et al.* Genome-wide association study identifies sequence variants on 6q21 associated with age at menarche. *Nat. Genet.* **41**, 734–8 (2009).

14. Ong, K. K. *et al.* Genetic variation in LIN28B is associated with the timing of puberty. *Nat. Genet.* **41**, 729–33 (2009).

15. Lindgren, C. M. *et al.* Genome-wide association scan meta-analysis identifies three Loci influencing adiposity and fat distribution. *PLoS Genet.* **5**, e1000508 (2009).

16. Nolte, I. M. *et al.* Common genetic variation near the phospholamban gene is associated with cardiac repolarisation: meta-analysis of three genome-wide association studies. *PLoS ONE* **4**, e6138 (2009).

17. Latourelle, J. C. *et al.* Genomewide association study for onset age in Parkinson disease. *BMC Med. Genet.* **10**, 98 (2009).

18. Chasman, D. I. *et al.* Genetic loci associated with plasma concentration of low-density lipoprotein cholesterol, high-density lipoprotein cholesterol, triglycerides, apolipoprotein A1, and Apolipoprotein B among 6382 white women in genome-wide analysis with replication. *Circ Cardiovasc Genet* **1**, 21–30 (2008).

19. Soranzo, N. *et al.* A genome-wide meta-analysis identifies 22 loci associated with eight hematological parameters in the HaemGen consortium. *Nat. Genet.* **41**, 1182–90 (2009).

20. Ferreira, M. A. R. *et al.* Sequence variants in three loci influence monocyte counts and erythrocyte volume. *Am. J. Hum. Genet.* **85**, 745–9 (2009).

21. Ganesh, S. K. *et al.* Multiple loci influence erythrocyte phenotypes in the CHARGE Consortium. *Nat. Genet.* **41**, 1191–8 (2009).

22. Repapi, E. *et al.* Genome-wide association study identifies five loci associated with lung function. *Nat. Genet.* **42**, 36–44 (2010).

23. Heid, I. M. *et al.* Genome-wide association analysis of high-density lipoprotein cholesterol in the population-based KORA study sheds new light on intergenic regions. *Circ Cardiovasc Genet* **1**, 10–20 (2008).

24. Ferreira, M. A. R. *et al.* Quantitative trait loci for CD4:CD8 lymphocyte ratio are associated with risk of type 1 diabetes and HIV-1 immune control. *Am. J. Hum. Genet.* **86**, 88–92 (2010).

25. Codd, V. *et al.* Common variants near TERC are associated with mean telomere length. *Nat. Genet.* **42**, 197–9 (2010).

26. Okada, Y. *et al.* Common variations in PSMD3-CSF3 and PLCB4 are associated with neutrophil count. *Hum. Mol. Genet.* **19**, 2079–85 (2010).

27. Medland, S. E. *et al.* A variant in LIN28B is associated with 2D:4D finger-length ratio, a putative retrospective biomarker of prenatal testosterone exposure. *Am. J. Hum. Genet.* **86**, 519–25 (2010).

28. Ramdas, W. D. *et al.* A genome-wide association study of optic disc parameters. *PLoS Genet.* **6**, e1000978 (2010).

29. Kang, T.-W. *et al.* Genome-wide association of serum bilirubin levels in Korean population. *Hum. Mol. Genet.* **19**, 3672–8 (2010).

30. Jee, S. H. *et al.* Adiponectin concentrations: a genome-wide association study. *Am. J. Hum. Genet.* **87**, 545–52 (2010).

31. Heid, I. M. *et al.* Meta-analysis identifies 13 new loci associated with waist-hip ratio and reveals sexual dimorphism in the genetic basis of fat distribution. *Nat. Genet.* **42**, 949–60 (2010).

32. Ikram, M. K. *et al.* Four novel Loci (19q13, 6q24, 12q24, and 5q14) influence the microcirculation in vivo. *PLoS Genet.* **6**, e1001184 (2010).

33. Paternoster, L. *et al.* Genome-wide association meta-analysis of cortical bone mineral density unravels allelic heterogeneity at the RANKL locus and potential pleiotropic effects on bone. *PLoS Genet.* **6**, e1001217 (2010).

34. Okada, Y. *et al.* Genome-wide association study for C-reactive protein levels identified pleiotropic associations in the IL6 locus. *Hum. Mol. Genet.* **20**, 1224–31 (2011).

35. Khor, C. C. *et al.* Genome-wide association studies in Asians confirm the involvement of ATOH7 and TGFBR3, and further identify CARD10 as a novel locus influencing optic disc area. *Hum. Mol. Genet.* **20**, 1864–72 (2011).

36. Kato, N. *et al.* Meta-analysis of genome-wide association studies identifies common variants associated with blood pressure variation in east Asians. *Nat. Genet.* **43**, 531–8 (2011).

37. Prescott, J. *et al.* Genome-wide association study of relative telomere length. *PLoS ONE* **6**, e19635 (2011).

38. Han, S. *et al.* Association of variants in FRAP1 and PDGFRA with corneal curvature in Asian populations from Singapore. *Hum. Mol. Genet.* **20**, 3693–8 (2011).

39. Kilpeläinen, T. O. *et al.* Genetic variation near IRS1 associates with reduced adiposity and an impaired metabolic profile. *Nat. Genet.* **43**, 753–60 (2011).

40. Okada, Y. *et al.* Identification of nine novel loci associated with white blood cell subtypes in a Japanese population. *PLoS Genet.* **7**, e1002067 (2011).

41. Schosser, A. *et al.* Genomewide association scan of suicidal thoughts and behaviour in major depression. *PLoS ONE* **6**, e20690 (2011).

42. Bis, J. C. *et al.* Meta-analysis of genome-wide association studies from the CHARGE consortium identifies common variants associated with carotid intima media thickness and plaque. *Nat. Genet.* **43**, 940–7 (2011).

43. Kim, Y. J. *et al.* Large-scale genome-wide association studies in East Asians identify new genetic loci influencing metabolic traits. *Nat. Genet.* **43**, 990–5 (2011).

44. Wain, L. V. *et al.* Genome-wide association study identifies six new loci influencing pulse pressure and mean arterial pressure. *Nat. Genet.* **43**, 1005–11 (2011).

45. Geller, F. *et al.* Genome-wide association study identifies four loci associated with eruption of permanent teeth. *PLoS Genet.* **7**, e1002275 (2011).

46. Lei, S.-F. *et al.* Genome-wide association study identifies HMGN3 locus for spine bone size variation in Chinese. *Hum. Genet.* **131**, 463–9 (2012).

47. Trompet, S. *et al.* Replication of LDL GWAs hits in PROSPER/PHASE as validation for future (pharmaco)genetic analyses. *BMC Med. Genet.* **12**, 131 (2011).

48. N'Diaye, A. *et al.* Identification, replication, and fine-mapping of Loci associated with adult height in individuals of african ancestry. *PLoS Genet.* **7**, e1002298 (2011).

49. Ohlsson, C. *et al.* Genetic determinants of serum testosterone concentrations in men. *PLoS Genet.* **7**, e1002313 (2011).

50. Carty, C. L. *et al.* Genome-wide association study of body height in African Americans: the Women's Health Initiative SNP Health Association Resource (SHARe). *Hum. Mol. Genet.* **21**, 711–20 (2012).

51. Stolk, L. *et al.* Meta-analyses identify 13 loci associated with age at menopause and highlight DNA repair and immune pathways. *Nat. Genet.* **44**, 260–8 (2012).

52. Naitza, S. *et al.* A genome-wide association scan on the levels of markers of inflammation in Sardinians reveals associations that underpin its complex regulation. *PLoS Genet.* **8**, e1002480 (2012).

53. Paternoster, L. *et al.* Genome-wide association study of three-dimensional facial morphology identifies a variant in PAX3 associated with nasion position. *Am. J. Hum. Genet.* **90**, 478–85 (2012).

54. Froguel, P. *et al.* A genome-wide association study identifies rs2000999 as a strong genetic determinant of circulating haptoglobin levels. *PLoS ONE* **7**, e32327 (2012).

55. Doumatey, A. P. *et al.* C-reactive protein (CRP) promoter polymorphisms influence circulating CRP levels in a genome-wide association study of African Americans. *Hum. Mol. Genet.* **21**, 3063–72 (2012).

56. Rawal, R. *et al.* Meta-analysis of two genome-wide association studies identifies four genetic loci associated with thyroid function. *Hum. Mol. Genet.* **21**, 3275–82 (2012).

57. Taal, H. R. *et al.* Common variants at 12q15 and 12q24 are associated with infant head circumference. *Nat. Genet.* **44**, 532–8 (2012).

58. Estrada, K. *et al.* Genome-wide meta-analysis identifies 56 bone mineral density loci and reveals 14 loci associated with risk of fracture. *Nat. Genet.* **44**, 491–501 (2012).

59. van Koolwijk, L. M. E. *et al.* Common genetic determinants of intraocular pressure and primary open-angle glaucoma. *PLoS Genet.* **8**, e1002611 (2012).

60. Luciano, M. *et al.* Genome-wide association uncovers shared genetic effects among personality traits and mood states. *Am. J. Med. Genet. B Neuropsychiatr. Genet.* **159B**, 684–95 (2012).

61. Kim, J. W. *et al.* A common variant in SLC8A1 is associated with the duration of the electrocardiographic QT interval. *Am. J. Hum. Genet.* **91**, 180–4 (2012).

62. Qi, Q. *et al.* Genome-wide association analysis identifies TYW3/CRYZ and NDST4 loci associated with circulating resistin levels. *Hum. Mol. Genet.* **21**, 4774–80 (2012).

63. Boraska, V. *et al.* Genome-wide association analysis of eating disorder-related symptoms, behaviors, and personality traits. *Am. J. Med. Genet. B Neuropsychiatr. Genet.* **159B**, 803–11 (2012).

64. Mangino, M. *et al.* Genome-wide meta-analysis points to CTC1 and ZNF676 as genes regulating telomere homeostasis in humans. *Hum. Mol. Genet.* **21**, 5385–94 (2012).

65. Liu, F. *et al.* A genome-wide association study identifies five loci influencing facial morphology in Europeans. *PLoS Genet.* **8**, e1002932 (2012).

66. Candille, S. I. *et al.* Genome-wide association studies of quantitatively measured skin, hair, and eye pigmentation in four European populations. *PLoS ONE* **7**, e48294 (2012).

67. Horikoshi, M. *et al.* New loci associated with birth weight identify genetic links between intrauterine growth and adult height and metabolism. *Nat. Genet.* **45**, 76–82 (2013).

68. Köttgen, A. *et al.* Genome-wide association analyses identify 18 new loci associated with serum urate concentrations. *Nat. Genet.* **45**, 145–54 (2013).

69. Dai, X. *et al.* A genome-wide association study for serum bilirubin levels and gene-environment interaction in a Chinese population. *Genet. Epidemiol.* **37**, 293–300 (2013).

70. Chu, A. Y. *et al.* Novel locus including FGF21 is associated with dietary macronutrient intake. *Hum. Mol. Genet.* **22**, 1895–902 (2013).

71. Paternoster, L. *et al.* Genetic determinants of trabecular and cortical volumetric bone mineral densities and bone microstructure. *PLoS Genet.* **9**, e1003247 (2013).

72. Chen, Z. *et al.* Genome-wide association analysis of red blood cell traits in African Americans: the COGENT Network. *Hum. Mol. Genet.* **22**, 2529–38 (2013).

73. Cousminer, D. L. *et al.* Genome-wide association and longitudinal analyses reveal genetic loci linking pubertal height growth, pubertal timing and childhood adiposity. *Hum. Mol. Genet.* **22**, 2735–47 (2013).

74. Hao, Y. *et al.* Genome-wide association study in Han Chinese identifies three novel loci for human height. *Hum. Genet.* **132**, 681–9 (2013).

75. Stambolian, D. *et al.* Meta-analysis of genome-wide association studies in five cohorts reveals common variants in RBFOX1, a regulator of tissue-specific splicing, associated with refractive error. *Hum. Mol. Genet.* **22**, 2754–64 (2013).

76. Trzaskowski, M. *et al.* First genome-wide association study on anxiety-related behaviours in childhood. *PLoS ONE* **8**, e58676 (2013).

77. Zheng, H.-F. *et al.* Meta-analysis of genome-wide studies identifies MEF2C SNPs associated with bone mineral density at forearm. *J. Med. Genet.* **50**, 473–8 (2013).

78. Demerath, E. W. *et al.* Genome-wide association study of age at menarche in African-American women. *Hum. Mol. Genet.* **22**, 3329–46 (2013).

79. Bae, H. T. *et al.* Genome-wide association study of personality traits in the long life family study. *Front Genet* **4**, 65 (2013).

80. Graff, M. *et al.* Genome-wide analysis of BMI in adolescents and young adults reveals additional insight into the effects of genetic loci over the life course. *Hum. Mol. Genet.* **22**, 3597–607 (2013).

81. Rietveld, C. A. *et al.* GWAS of 126,559 individuals identifies genetic variants associated with educational attainment. *Science* **340**, 1467–71 (2013).

82. Sim, X. *et al.* Genetic loci for retinal arteriolar microcirculation. *PLoS ONE* **8**, e65804 (2013).

83. Liu, C.-T. *et al.* Genome-wide association of body fat distribution in African ancestry populations suggests new loci. *PLoS Genet.* **9**, e1003681 (2013).

84. Himes, B. E. *et al.* ITGB5 and AGFG1 variants are associated with severity of airway responsiveness. *BMC Med. Genet.* **14**, 86 (2013).

85. Kleber, M. E. *et al.* Genome-wide association study identifies 3 genomic loci significantly associated with serum levels of homoarginine: the AtheroRemo Consortium. *Circ Cardiovasc Genet* **6**, 505–13 (2013).

86. O'Seaghdha, C. M. *et al.* Meta-analysis of genome-wide association studies identifies six new Loci for serum calcium concentrations. *PLoS Genet.* **9**, e1003796 (2013).

87. Cheng, C.-Y. *et al.* Nine loci for ocular axial length identified through genome-wide association studies, including shared loci with refractive error. *Am. J. Hum. Genet.* **93**, 264–77 (2013).

88. Yatagai, Y. *et al.* Genome-wide association study for levels of total serum IgE identifies HLA-C in a Japanese population. *PLoS ONE* **8**, e80941 (2013).

89. Moayyeri, A. *et al.* Genetic determinants of heel bone properties: genome-wide association meta-analysis and replication in the GEFOS/GENOMOS consortium. *Hum. Mol. Genet.* **23**, 3054–68 (2014).

90. Nongpiur, M. E. *et al.* ABCC5, a gene that influences the anterior chamber depth, is associated with primary angle closure glaucoma. *PLoS Genet.* **10**, e1004089 (2014).

91. An, P. *et al.* Genome-wide association studies identified novel loci for non-high-density lipoprotein cholesterol and its postprandial lipemic response. *Hum. Genet.* **133**, 919–30 (2014).

92. Kwak, S. H. *et al.* A genome-wide association study on thyroid function and anti-thyroid peroxidase antibodies in Koreans. *Hum. Mol. Genet.* **23**, 4433–42 (2014).

93. Loth, D. W. *et al.* Genome-wide association analysis identifies six new loci associated with forced vital capacity. *Nat. Genet.* **46**, 669–77 (2014).

94. Arking, D. E. *et al.* Genetic association study of QT interval highlights role for calcium signaling pathways in myocardial repolarization. *Nat. Genet.* **46**, 826–36 (2014).

95. Chen, P. *et al.* CMPK1 and RBP3 are associated with corneal curvature in Asian populations. *Hum. Mol. Genet.* **23**, 6129–36 (2014).

96. Bolton, J. L. *et al.* Genome wide association identifies common variants at the SERPINA6/SERPINA1 locus influencing plasma cortisol and corticosteroid binding globulin. *PLoS Genet.* **10**, e1004474 (2014).

97. Ramirez, A. *et al.* SUCLG2 identified as both a determinator of CSF AB1-42 levels and an attenuator of cognitive decline in Alzheimer's disease. *Hum. Mol. Genet.* **23**, 6644–58 (2014).

98. Hong, K.-W. *et al.* Identification of three novel genetic variations associated with electrocardiographic traits (QRS duration and PR interval) in East Asians. *Hum. Mol. Genet.* **23**, 6659–67 (2014).

99. Li, J. *et al.* Genome-wide admixture and association study of serum iron, ferritin, transferrin saturation and total iron binding capacity in African Americans. *Hum. Mol. Genet.* **24**, 572–81 (2015).

100. Lüneburg, N. *et al.* Genome-wide association study of L-arginine and dimethylarginines reveals novel metabolic pathway for symmetric dimethylarginine. *Circ Cardiovasc Genet* **7**, 864–72 (2014).

101. Lu, X. *et al.* Genome-wide association study in Chinese identifies novel loci for blood pressure and hypertension. *Hum. Mol. Genet.* **24**, 865–74 (2015).

102. van der Valk, R. J. P. *et al.* A novel common variant in DCST2 is associated with length in early life and height in adulthood. *Hum. Mol. Genet.* **24**, 1155–68 (2015).

103. Wood, A. R. *et al.* Defining the role of common variation in the genomic and biological architecture of adult human height. *Nat. Genet.* **46**, 1173–86 (2014).

104. Kim, S., Shin, C. & Jee, S. H. Genetic variants at 1q32.1, 10q11.2 and 19q13.41 are associated with prostate-specific antigen for prostate cancer screening in two Korean population-based cohort studies. *Gene* **556**, 199–205 (2015).
